# Focus on Osteosclerotic Progression in Primary Myelofibrosis

**DOI:** 10.3390/biom11010122

**Published:** 2021-01-19

**Authors:** Mariarita Spampinato, Cesarina Giallongo, Alessandra Romano, Lucia Longhitano, Enrico La Spina, Roberto Avola, Grazia Scandura, Ilaria Dulcamare, Vincenzo Bramanti, Michelino Di Rosa, Nunzio Vicario, Rosalba Parenti, Giovanni Li Volti, Daniele Tibullo, Giuseppe A. Palumbo

**Affiliations:** 1 Section of Biochemistry, Department of Biomedical and Biotechnological Sciences, University of Catania, 95123 Catania, Italy; mariaritaspampinato93@gmail.com (M.S.); lucialonghitano@icloud.com (L.L.); ravola@unict.it (R.A.); d.tibullo@unict.it (D.T.); 2 Department of Medical and Surgical Sciences and Advanced Technologies “G.F. Ingrassia”, University of Catania, 95123 Catania, Italy; cesarina.giallongo@unict.it; 3 Department of General Surgery and Medical-Surgical Specialties, Division of Hematology, A.O.U. “Policlinico-Vittorio Emanuele”, University of Catania, 95123 Catania, Italy; sandrina.romano@gmail.com (A.R.); enricolaspina@outlook.it (E.L.S.); gra.scandura@gmail.com (G.S.); dulcamareilaria@gmail.com (I.D.); 4 Division of Clinical Pathology, “Giovanni Paolo II” Hospital–A.S.P. Ragusa, 97100 Ragusa, Italy; vincenzo.bramanti@asp.rg.it; 5 Section of Human Anatomy and Histology, Department of Biomedical and Biotechnological Sciences, University of Catania, 95123 Catania, Italy; chitotriosidase@gmail.com; 6 Section of Physiology, Department of Biomedical and Biotechnological Sciences, University of Catania, 95123 Catania, Italy; nunzio.vicario@unict.it (N.V.); parenti@unict.it (R.P.)

**Keywords:** primary myelofibrosis, bone, myeloproliferative neoplasm, bone marrow, fibrosis

## Abstract

Primary myelofibrosis (PMF) is a myeloproliferative neoplasm characterized by hematopoietic stem-cell-derived clonal proliferation, leading to bone marrow (BM) fibrosis. Hematopoiesis alterations are closely associated with modifications of the BM microenvironment, characterized by defective interactions between vascular and endosteal niches. As such, neoangiogenesis, megakaryocytes hyperplasia and extensive bone marrow fibrosis, followed by osteosclerosis and bone damage, are the most relevant consequences of PMF. Moreover, bone tissue deposition, together with progressive fibrosis, represents crucial mechanisms of disabilities in patients. Although the underlying mechanisms of bone damage observed in PMF are still unclear, the involvement of cytokines, growth factors and bone marrow microenvironment resident cells have been linked to disease progression. Herein, we focused on the role of megakaryocytes and their alterations, associated with cytokines and chemokines release, in modulating functions of most of the bone marrow cell populations and in creating a complex network where impaired signaling strongly contributes to progression and disabilities.

## 1. Introduction

Primary myelofibrosis (PMF), known as a clonal stem cell disorder, is a chronic myeloproliferative syndrome representing the rarest and most complex of all BCR-ABL-negative myeloproliferative neoplasms (MPNs), a group of neoplastic hematological diseases comprising essential thrombocythemia (ET) and polycythemia vera (PV). From a genetic point of view, the vast majority of patients show the JAK2V617F driver mutation; the remaining population of patients usually shows either calreticulin (CALR) mutations or thrombopoietin receptor mutations (i.e., myeloproliferative leukemia, MPL) [1,2,3,4]. These mutations could also be associated with concomitant mutations to other genes such as ASXL1, IDH1/2, EZH2, DNMT3A and SRSF2 [4,5,6] and with microRNA expression level alterations [7]. The main mutations show similarity in the constitutive activation of JAK/STAT signaling. This overactivation represents a critical feature of clonal myelopoiesis in MPNs and the main biochemical pathway involved in the pathogenetic progression of myelofibrosis [8,9], playing a role in malignant expansion and compromising nonclonal hematopoietic bone marrow cell populations [10,11]. In addition to JAK/STAT, the involvement and hyperactivation of PI3K/AKT and NF-kB pathways in MPN disorders have been reported [12,13]. Pathological onset is due to the neoplastic transformation of a multipotent hematopoietic stem cell in the bone marrow niche and the subsequent proliferation of newly formed clones with a cancer outbreak. Therefore, as a consequence of the pathological upsurge, the bone marrow environment undergoes morphological and functional changes, inducing abnormalities in granulocytes, megakaryocytes and stromal cells, such as fibroblasts. Indeed, megakaryocytes show reduced GATA-1 protein expression coupled with increased levels of many inflammatory cytokines and growth factors (b-FGF, VEGF, PDGF) in addition to extracellular matrix constituents including fibronectin, reticulin and collagens. Disrupted cell interactions and functional variations of stroma, BM-MSC, megakaryocytes, osteoblasts, endothelium and myofibroblasts culminate in the development of bone marrow damage with an inflammatory and profibrotic environment [7].

## 2. Clinical Features

Overt PMF is the least common [14] of MPN disorders, associated with dismal prognosis with an estimated survival of 2–5 years postdisease onset, slightly improved with current therapeutic approaches and cured with allogeneic stem cell transplantation [15,16,17]. The most common clinical hallmarks of PMF range from constitutional symptoms (fatigue, cachexia) to symptomatic anemia, thrombohemorrhagic events, hepatosplenomegaly with extramedullary hematopoiesis and increased susceptibility to infections and secondary cancers [18,19,20,21]. PMF is also considered a model where the neoplastic condition is present together with an elevated inflammatory status, and new therapies seem to target both aspects [22]. Bone marrow fibrosis is a key characteristic of the disease and is outlined by abnormal trafficking patterns between stem cells, hematopoietic progenitors and cell lineages in the cancer microenvironment. As a consequence, hematopoietic cells migrate from the bone marrow and give rise to extramedullary hematopoiesis, with erythroid and myeloid progenitors outside the primary niche. Hence, cancer cells migrate to external sites, such as the liver or spleen, and the expansion of the malignant clones leads to progressive hepatosplenomegaly [23]. Splenomegaly represents an unavoidable outcome and may lead to clinical complications such as splenic infarction, hemorrhages or thrombosis [14,24,25,26,27], with a severe impact on prognosis.

### Bone Marrow Niche and Microenvironment Disruptions in PMF

**Figure 1:**
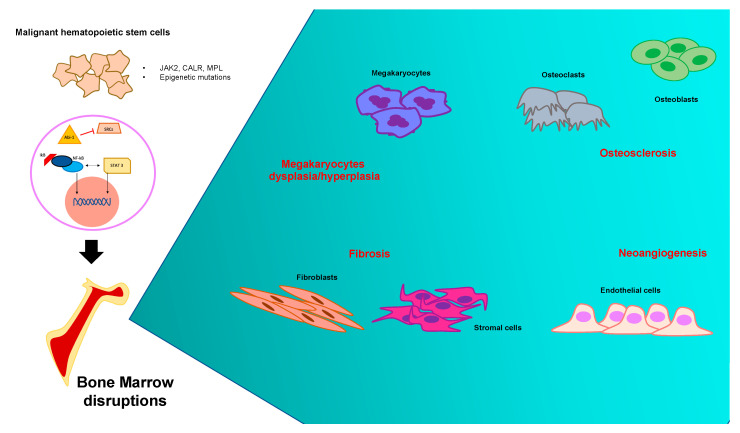
Pathogenetic mechanisms of primary myelofibrosis and cell lines involved: summary panel. Bone marrow dysregulations due to the neoplastic expansion of one hematopoietic stem cell produce a number of events that took place within the pathological microenvironment. The most relevant clinical consequences are neoangiogenesis, megakaryocytes hyperplasia and extensive bone marrow fibrosis, followed by osteosclerosis and bone damage.

Bone marrow represents a complex and heterogenous microenvironment in which physiological homeostasis and cellular activities are based on continuous crosstalk between hematopoietic and stromal niches, in close communication throughout environmental signals, growth factors, adhesion molecules and the vascular network. The stem area is composed of stem precursors and endosteal bone surface, in which resident hematopoietic stem cells (HSCs) proliferate and differentiate [28]. Osteoblastic and vascular compartments are characterized by a heterogenous group of cells such as hematopoietic cells, fibroblasts, osteoblasts and osteoclasts, adipocytes, stromal cells (vascular endothelial-cadherin-positive sinusoidal endothelial cells (SECs)), perivascular cells and mesenchymal stem cells (MSCs) [29]. In such a complex microenvironment, extracellular matrix (ECM) elements provide both mechanical and functional support [30]. The physiological mechanisms of proliferation and differentiation of HSCs are strictly dependent on homeostatic communication between bone marrow compartments. A functional imbalance of the stromal niche is considered the major factor in inducing the clinical consequences in PMF. In synthesis, it is believed that bone marrow impairment is due to multifactorial damage involving several cell types and the dysregulations and/or dysfunctions of biochemical elements (Figure 1).

## 3. Fibrosis as PMF Banner

PMF complexity is mainly due to the onset of bone marrow fibrosis, followed by a long sequence of cascade events. This results in hematopoiesis impairment and organ failure, culminating in osteosclerotic deposition during the late stage of the disease, which seriously impairs the health of affected patients [31]. Thus, fibrosis represents one of the cardinal hallmarks of pathological progression in PMF [32]. It seems to be mainly promoted by transforming growth factor β (TGF-β), matrix metalloproteinase-9 (MMP-9) and tissue inhibitor of metalloproteinases (TIMPs) [33,34], but increased expression of growth factors such as osteocalcin, b-fibroblast growth factor (b-FGF), platelet-derived growth factor (PDGF) and vascular endothelial growth factor (VEGF) has been reported [35]. This heterogenous group of biomarkers has detrimental impacts on vascularization, the MSC niche and ECM components’ stability [36]. Bone marrow fibrosis occurs as a cytokine-mediated secondary reaction toward the starting clonal malignant expansion [37,38] and is characterized by disproportionate deposits of ECM proteins [39]. In vivo and in vitro studies have clarified the role of several cytokines in the aberrant stromal reaction, with a strong emphasis on the pleiotropic cytokine TGF-β [40,41,42]. Analysis of biochemical markers in PMF patients showed that procollagen type 1 N-terminal propeptide (P1NP) was significantly increased, most likely reflecting the relevant collagen deposition in bone marrow due to disease progression [43]. Fibroblast stimulation, as well as megakaryocytes activation, elicits TGF-β release, which induces large amounts of ECM proteins and cell adhesion molecules while enhancing the expression of inhibitory proteases involved in the degradation of the ECM [44]. At this point, it is easy to hypothesize a backdrop in which the microenvironment itself induces severe inflammation with consequent acidification of the medullary site [45]. This pathological picture influences HSCs and MSCs, which are subjected to clonal neoplastic expansion and subsequent differentiation into monocytes and megakaryocytes. The latter population releases a large number of inflammatory cytokines giving rise to a robust inflammatory cascade, responsible for the permanent alteration of the hematopoietic niche, altered crosstalk between cells and severe hematopoietic deficit [46].

## 4. Megakaryocytes Role in Bone Marrow Imbalance

Altered megakaryocytes produce a plethora of growth factors, interleukins and cytokines involved in the onset of a dysfunctional microenvironment, contributing to the neoangiogenesis and hyperactivation of both fibroblasts and osteoblasts [47]. The functional role of abnormal megakaryocytes in PMF has been investigated through different approaches, using both in vitro and in vivo models coupled with evidence from pathological assessments of clinical samples [33,34,48,49]. Histological analysis of bone marrow biopsies from PMF patients revealed megakaryocytic clusters and hyperplasia [50]. In particular, evidence from knockout mice models showed that GATA1 or the thrombopoietin (TPO) receptor, which are physiologically involved in megakaryocyte maturation, are involved in altered megakaryocytes development as well as reduced platelet counts [34,48]. In addition, TPO receptor overexpression gives rise to megakaryocytic and granulocytic hyperplasia with erythroblasts hypoplasia resulting in a fatal myeloproliferative condition [33,49]. In this regard, megakaryocyte hyperplasia has been investigated in in vitro models of CD34-positive cells isolated from PMF patients cocultured with TPO and stem-cell-derived factors. This evidence shows that CD34-positive progenitor cells give rise to megakaryocytes with impaired apoptosis due to the overexpression of the antiapoptotic protein Bcl-xL, which promotes cellular hyperplasia [7]. Accumulating evidence particularly supports a potential role of IL-8 and its CXCR1 and CXCR2 receptors, both belonging to the G protein-coupled receptor superfamily, on the dysfunctional phenotype of megakaryocytes. IL-8 acts as a chemoattractive and proinflammatory agent as well as a neutrophil activator, interacting with the abovementioned receptors [51]. Many different cell types are involved in IL-8 secretion, such as macrophages, fibroblasts, monocytes and megakaryocytes, at least in in vitro settings [52,53,54]. Hence, this cytokine and CXCRs in megakaryocytes dysfunction play a key role in this setting. In particular, IL-8 levels have been found to be significantly increased in PMF patient serum and, in addition, CXCR1 and CXCR2 participate in megakaryocyte proliferation and megakaryocyte ploidy [51]. In this regard, it is known that PMF patients exhibit aberrant immature megakaryocyte clusters, releasing a plethora of proinflammatory cytokines, a key mechanism that induces secondary fibrosis [33]. Much evidence suggests that the enhancement of the role of the FL/Flt3 axis in PMF could be associated with dysmegakaryopoiesis, as shown by an increased percentage of circulating CD34^+^Flt3^+^ cells expressing the CD41 megakaryocyte antigen [55]. Among the most important chemokines, CXCL4, also known as platelet factor-4 (PF4), was proposed to play a crucial role in PMF pathogenesis in 1984 [56]. However, the underlying mechanism of action of CXCL4, produced by the clonal pathological hematopoietic stem cell, has been recently elucidated [57]. In fact, CXCL4 is able to reprogram GLI1, upregulating matrisome genes, a prelude to fibrosis development. Furthermore, in MPN mouse models, CXCL4 knockdown prevents the upregulation of inflammatory pathways and TGF-beta, improving most of the main clinical signs observed in such a preclinical model such as anemia, thrombocytosis, splenomegaly and aberrant megakaryocytes in bone marrow. Finally, the upregulation of the JAK/STAT pathway induced by CXCL4 has been also reported, even though it seems this is not sufficient to develop fibrosis, whereas other mechanisms might coexist and cooperate.

## 5. The Biochemical Network of Osteosclerosis in PMF

Bone modifications are a pathognomonic hallmark of PMF since they represent one of the direct results of bone marrow disruption. Osteosclerosis remains the most common bone change, which represents a pathological event characterized by increased bone density and abnormal hardening [58,59,60], and its pathogenesis is still largely unknown. Osteosclerotic regions are produced by the irregular thickening of bone trabeculae, new bone shaping and consequent bone volume growth. In particular, increased bone marrow activity in some regions, such as the vertebral column, pelvis or proximal segments of long bones, remain the most affected by such alterations [58,60]. The physiological bone morphology and functionality are strictly dependent on the accurate setting of the marrow osteoblastic niche as well as the balance between mature bone tissue, endosteum and central bone marrow [61,62].

### 5.1. Bone Marrow as Bone Remodeling “Workshop”

Bone tissue homeostasis is controlled by the cooperation of both HSCs and MSCs, involved in the differentiation in osteoclasts (OCs) and osteoblasts (OBs), respectively. OBs share their mesenchymal biogenesis with chondrocytes, adipocytes and stromal cells [63,64,65,66]. As such, bone marrow hematopoiesis and bone turnover have a morphological and functional interconnection, and both these processes affect each other. The osteoblastic niche holds different cell lineages that support HSC multipotency and self-renewal through reciprocal interactions, including bone-matrix-forming OBs and bone-resorbing OCs [67]. The development of OCs from HSCs provides a first step of monocytes/macrophage differentiation as progenitor cells, followed by the subsequent formation of mononuclear OCs. Although these cells already show bone-resorbing activity, they subsequently fuse to produce multinuclear osteoclasts in order to perform their specific functions toward bone remodeling [68]. Conversely, OBs originate from mesenchymal-lineage MSCs and undergo two different processes: they become quiescent cells on the bone surface, known as the bone lining cell, or they differentiate into mature osteocytes [69,70]. Spindle-shaped N-cadherin-expressing osteoblasts (SNOs), a subset of osteoblastic lining cells in the trabecular bone area, prevent the differentiation process of HSCs, keeping them long-term quiescent. These resting cells coexist with the activated HSCs, which are recruited to differentiate from the vascular niche in response to microenvironmental changes [62]. In this regard, activated OBs regulate HSC quiescence through the secretion of angiopoietin-1 (Ang 1) and osteopontin (OPN) [71]; at the same time, OCs release calcium during bone resorption in order to contribute to enhancing HSC localization into bone marrow [72]. Similarly, CD146-positive OB progenitor subendothelial stromal cells are regarded as a critical component of the endosteal HSC niche and contribute to the organization and structure of sinusoidal walls, expressing HSC regulators such as Ang-1 or CXCL12 [73]. In fact, while staying over sinusoids, they contribute to hematopoietic regulation, acting either directly as adventitial reticular cells or indirectly through their OB progeny at the endosteal surface [74]. Notably, CD146 or melanoma cell adhesion molecule (MCAM) has been associated with the late stage of the disease. In particular, a remarkable increase in CD146 expression in patients during the advanced phase of PMF has been reported [75]. A recent study confirmed the importance of the endosteal niche in HSC maintenance, assuming an interesting model of mutual interaction between aberrant myeloid cells caused by myeloproliferative expansion, MSC stimulation and OB overproduction [76].

### 5.2. The OB/OC Ratio

It is well established that osteosclerotic evolution in PMF is mainly due to the failure of the bone formation and bone resorption balance, and OBs and OCs represent the main characters of osteosclerosis pathogenesis. On the one hand, OBs are strongly induced to proliferate and differentiate [77]; on the other hand, osteoclastogenesis seems to be deeply impaired as a result of microenvironmental alterations involving osteoprotegerin (OPG), RANKL and macrophage colony-stimulating factor (M-CSF) expressions. In particular, recent evidence suggests that in PMF patients, OCs are generated by neoplastic monocytes after a low number of fusion events, providing an abnormal morphology and impaired resorption capacity [78]. In this context, bone regeneration overcomes the bone resorption process, resulting in osteosclerotic deposition. OB enhancement certainly represents a distinctive feature of osteosclerotic development in PMF [76,79], and this evidence is followed by concurrent OC impairment, which further exacerbates the severe imbalance toward bone remodeling [78]. However, while several data support the idea of maintained OB–OC coupling, decreased bone resorption suggests that the individual activity of OCs, similar to that of OBs, could also be decreased. The decline in OC activity is recognized as a positive balance by remodeling bone units, which could possibly lead to the growth of bone mass in patients [80]. OBs, in turn, are stimulated by excessive bone morphogenetic proteins BMP-2, -4 and -6, mainly released by abnormal megakaryocytes [81] and also by growth factors able to induce their proliferation and differentiation, such as insulin-like growth factor I (IGF-I) and fibroblast growth factor (FGF) [77]. BMPs belong to the TGF-β superfamily, and their release is associated with increased gene expression of type I collagen, osterix (Osx), osteocalcin, osteopontin (OPN), VEGF and PDGFα during osteoblastic differentiation [82]. Their involvement in neoangiogenesis and osteosclerosis, upregulating OB proliferation and differentiation, has been hypothesized on the basis of experimental evidence showing that megakaryocytes of GATA-1-low mice contribute to osteosclerosis by stimulating bone formation via the increased release of BMPs [83]. Moreover, their pathogenetic function in PMF seems to be correlated with alterations in the NOG gene encoding for the antagonist protein to BMP2 and BMP4 (NOGGIN) [82,84].

### 5.3. The Monocytic Line: Role of Osteal Macrophages (OsteoMacs)

Osteosclerosis associated with MPNs is a pathological evolution, typical of the advanced stage of myelofibrosis, which is due to the gradual replacement of marrow by collagen and bone trabeculae accomplished by activated myofibroblasts (αSMA1-positive stromal cells) [85]. These types of cells differentiate by particular progenitors such as GLI1-positive and Lepr-positive stromal cells under the driving activity of megakaryocytes [86,87,88]. Many studies have revealed the importance of the monocyte line [89,90,91], highlighting the role of IL-1 release as well as TGFβ overproduction. In particular, bone-resident macrophages (OsteoMacs) have recently gained relevance in PMF clinical contexts because of their involvement in the differentiation of mesenchymal lineages [92,93,94], especially in OB functionality, through TNFα or oncostatin M [95,96]. It is well known that OsteoMacs contribute to bone repair mechanisms; after a proinflammatory stimulus, they release IL1 and TNFα [97] and also secrete proanabolic factors to support osteogenic differentiation and OB maturation in vitro [95,98,99,100], closely cooperating with MSCs and stromal cells to form a deep partnership with the neighboring populations. Starting with this evidence, some studies confirmed the implication of the bone-associated macrophage lineage in the myelofibrotic and osteosclerotic course through a complex release of growth factors such as TGFβ, CXCL4 and PDGF, in collaboration with megakaryocytes, as mentioned above. In this backdrop, the mutual regulation between megakaryocytes and macrophages in MPN progression has been hypothesized, and vitamin D seems to play a pivotal role in their crosstalk [101].

### 5.4. RANKL/OPG Axis and the Wnt/b-Catenin Pathway

**Figure 2:**
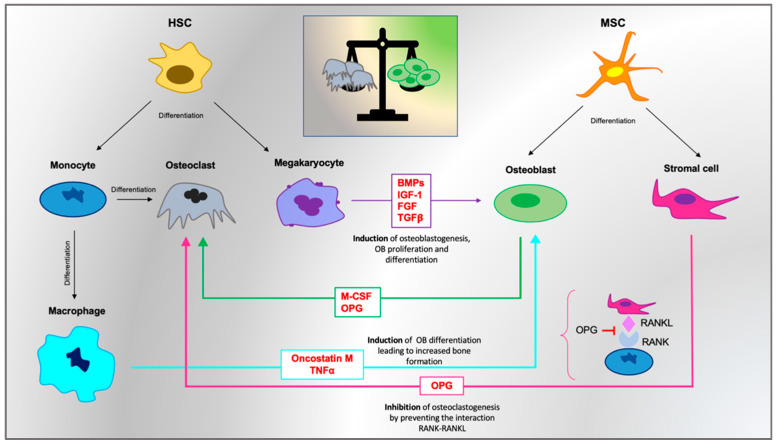
A schematic representation of osteosclerosis processes in primary myelofibrosis (PMF).

Much evidence has been reported to support the role of osteoprotegerin (OPG) as a substantial marker involved in PMF pathogenesis [102,103]. Plasma OPG levels have been found significantly increased in PMF patients compared to healthy controls. OPG, a member of the TNFr superfamily [104], constitutes a key biomolecule in the bone remodeling process, regulating the inhibition of OC differentiation. Its up- or downregulation is involved in different pathological conditions associated with osteosclerosis [105] or osteoporosis [106,107], respectively. OPG hyperexpression particularly seems to support a double function. On the one hand, it impairs OC production and its differentiation process [108]; on the other hand, it sustains endothelial proliferation as well as neoangiogenesis [109,110]. OPG expression in OBs is regulated by many different cytokines, as well as by the Wnt/βcatenin pathway [111] and Jagged1/Notch1 signaling, which directly inhibits osteoclastogenesis and indirectly affects the OPG-receptor activator of the NF-kB ligand (RANKL) expression ratio in stromal cells [112]. The main signaling pathway in bone resorption is indeed RANKL mediated. It is mainly expressed by osteoblastic stromal cells that bind to its receptor RANK on monocytes, OCPs and mature OCs, producing osteoclastogenesis [113,114]. RANKL is a homotrimeric protein existing like membrane-bound OBs in a T cell form or secretory protein form [115,116]. Increased RANKL expression in stromal cells is usually associated with the stimulation of osteoclastogenesis and OC progenitor (OCP) release [117,118,119]. Furthermore, RANKL represents a functional link between bone remodeling and hematopoiesis since RANKL-induced osteoclastogenesis affects HSC mobilization as well as hematopoietic activity [117]. Experimental evidence highlights a role of TGFβ on osteosclerotic progression together with OPG upregulation [105], while the stimulatory effect of TGFβ on OPG secretion in primary OBs and stromal cell lines has been confirmed by many other studies [120,121]. The RANKL/OPG ratio is an essential factor of bone mass regulation and integrity. In particular, OPG represents an inhibitor of bone resorption and protects bone binding to RANKL, impeding interaction to its receptor RANK (Figure 2). The canonical WNT/β-catenin signaling pathway plays an important role during skeleton development, besides being important for bone mass. WNT signaling also regulates MSC differentiation into OBs, controlling bone formation, increasing OB proliferation and inhibiting OB apoptosis. It is also able to negatively regulate adipocyte, chondrocyte and OC differentiation. The canonical WNT/β-catenin signaling is a key mediator of the stem cell signaling network, in which different cytokine-induced cascades act in a context-dependent manner [122]. A major protagonist of this network is Wnt, a factor of fibroblast growth (FGF), Notch, transforming growth factor b/bone morphogenetic protein (TGF-b/BMP) and sonic hedgehog signaling (SHH) cascades [123,124]. All these pathways promote bone remodeling, inducing MSC differentiation into mature OBs. Several works have demonstrated that WNT/β-catenin signaling is involved in microenvironmental transformation in PMF [125]. Moreover, the SHH pathway has been found to be upregulated in PMF, suggesting a potential interplay with WNT/β-catenin in mediating osteosclerotic mechanisms [126]. Recently, Yachoui and collaborators highlighted the role of endothelin-1 (ET1), a potent vasoconstrictor, as a key mediator of osteoblastic bone metastases by stimulating OB proliferation and new bone formation. The anabolic action of ET1 occurs through the activation of the WNT signaling pathway, reducing the expression of both DKK1 and SOST (inhibitors of canonical WNT signaling) and inducing the formation of new bone. These authors also demonstrated that PMF patients showed increased ET1 signaling, suggesting that it could be responsible for the osteosclerosis that developed with advanced myelofibrosis [127].

## 6. Conclusions

PMF is a myeloproliferative syndrome with a very complex clinical background, characterized by general bone marrow failure with impaired hematopoiesis followed by extramedullary hematopoiesis, splenomegaly and progressive bone deposition replacing the fibrotic areas. Bone tissue deposition remains one of the mechanisms, together with fibrosis, associated with unavoidable progression. Although several mechanisms of PMF bone damage remain unknown, the involvement of many biomarkers and cell lineages constitutively present in the bone marrow microenvironment has been confirmed. Assuming that the physiological state of bone marrow is based on the continuous balance between the hematopoietic niche and bone remodeling, it is necessary to evaluate the most important factors involved in bone impairment and osteosclerosis. In particular, we summarized the central role of megakaryocytes and their alterations associated with the release of a plethora of cytokines and chemokines. Each of them represents a relevant factor, the activity of which seems to be absolutely interconnected with each bone marrow cell population, creating a network of impaired signaling that contributes to the overall imbalance of the bone marrow system. In this complex system, each cell line, associated with a specific panel of cytokines and signals, contributes to bone alterations and modifies the physiological functions, affecting the fine balance between OBs and OCs and, in turn, affecting bone formation and bone resorption homeostasis.
